# An Analysis of Major Adverse Cardiovascular Events, Other Adverse Events, and Efficacy in Patients with Rheumatic Disease Receiving Targeted Therapy: Experience from a Third-Level Hospital

**DOI:** 10.3390/jcm14134693

**Published:** 2025-07-02

**Authors:** Marta Rojas-Giménez, Paloma Muñoz-Reinoso, María Dolores Arcila-Durán, Virginia Moreira-Navarrete, Manuel Maqueda López, María Dolores Fernández-Alba, Rafael Ariza-Ariza, Maria Daniela Decan-Bardasz, Blanca Hernández Cruz, Francisco Javier Toyos, Dolores Virginia Mendoza Mendoza, José Javier Pérez Venegas

**Affiliations:** Rheumatology Department, Virgen Macarena University Hospital (HUVM), 41009 Seville, Spain

**Keywords:** rheumatoid arthritis, cardiovascular risk, inflammatory activity, jakinibs

## Abstract

**Objectives:** We wished to evaluate the safety profile of the Janus kinase (JAK) inhibitors used in the Spanish population; to study the onset of major adverse cardiovascular events (MACEs) and thrombotic events (arterial and venous); and to analyze the factors associated with the onset of these events. **Methods:** We conducted a retrospective observational study of a cohort of patients with rheumatoid arthritis (RA), spondyloarthritis (SpA), and psoriatic arthritis (PsA) included in the biological therapy registry of the Rheumatology Department of Virgen Macarena University Hospital (HUVM), Seville, Spain, who started targeted treatment between 2019 and late 2024. We collected data on disease activity, traditional cardiovascular risk factors, the Charlson comorbidity index, previous synthetic or biologic drug therapy, the use of corticosteroids (and their dose), severity data (structural damage, extra-articular manifestations), and adverse events at the end of follow-up (e.g., MACEs, infections, neoplasms, and herpes zoster). We performed a descriptive bivariate analysis and a multivariate logistic regression analysis (dependent variable: MACEs) to identify factors that were independently associated with MACEs. **Results:** The study population comprised 137 patients (110 with RA, 18 with PsA, and 9 with SpA) who were followed up for a mean of 3.9 (2.6) years. Most patients had received JAK inhibitors as their second-line or subsequent treatment. At the end of the follow-up, 82 patients (66.7%) continued their treatment. Nine patients (6.6%) experienced a MACE, and five experienced a heart attack. All of these patients had RA. We found no differences between JAK inhibitors in terms of the incidence of the adverse events studied. Patients who experienced MACEs were more often male and smokers (current or former) and more often had hypertension and diabetes. No significant differences were found in the association with disease activity or previous or concomitant treatment. The factors that were independently associated with MACEs were a previous cardiovascular event (OR, 10.74; 95%CI, 1.05–113.7; *p* = 0.036), male sex (OR, 9.7; 95%CI, 1.6–76.5; *p* = 0.016), diabetes mellitus (OR, 10.3; 95%CI, 1.75–83; *p* = 0.013), and the duration of treatment with JAK inhibitors (OR, 1.47; 95%CI, 1.13–2.01; *p* = 0.005). **Conclusions:** We found no differences in the onset of adverse events, specifically MACEs, between the different JAK inhibitors analyzed. These events are more common in patients who already have cardiovascular risk factors, such as diabetes mellitus, or who have already experienced a cardiovascular event. JAK inhibitors broadly suppress cytokines in patients whose disease is refractory to other treatments. However, we must continue to evaluate their long-term safety in real-world studies.

## 1. Introduction

Patients with rheumatoid arthritis (RA), spondyloarthritis (SpA), and psoriatic arthritis (PsA) have a high cardiovascular risk, which is associated with increased mortality [1,2,3]. This is due to accelerated atherogenesis [4,5], which is affected by both traditional and non-traditional cardiovascular risk factors (CVRFs) [6,7,8,9,10].

Treatment with biologic disease-modifying antirheumatic drugs (bDMARDs) and targeted synthetic DMARDs significantly improves symptoms in patients who do not respond to standard treatment, such as conventional synthetic DMARDs (csDMARDs) [11]. The four currently available targeted treatments, namely the Janus kinase (JAK) inhibitors tofacitinib, baricitinib, filgotinib, and upadacitinib, present multiple immunomodulatory effects through the inhibition of JAKs, although their selective inhibition of different JAKs varies depending on the drug. Oral administration of JAK inhibitors has proven advantageous by increasing adherence and patient satisfaction. Moreover, they have proven efficacious in patients who do not respond to TNF-α inhibitors and are an additional option for disease control in difficult-to-treat patients [12]. The efficacy of JAK inhibitors in rheumatic diseases is well established, although in recent years, associated adverse events have given cause for concern. A non-inferiority post-authorization trial with tofacitinib found the risk of malignant cancer and major cardiovascular events (MACEs) to be higher than that for TNF-α inhibitors [13]. Consequently, the Pharmacovigilance Risk Assessment Committee (PRAC) of the European Medicines Agency (EMA) released a report in October 2022 concluding that these safety findings applied to all approved uses of JAK inhibitors in chronic inflammatory diseases and providing recommendations for their use in affected patients [14].

Randomized controlled clinical trials and their extension studies recently began to shed light on the safety profile of JAK inhibitors. However, we still lack in-depth knowledge of the safety profile of this treatment in rheumatic disease in clinical practice and of the different JAK inhibitors themselves [15].

The main objectives of the present study were to evaluate the safety profile of JAK inhibitors in our setting, study the onset of MACEs and thrombotic events (arterial and venous), analyze the factors associated with the onset of these events, evaluate differences between groups, and analyze other adverse events such as neoplasms.

## 2. Patients and Methods

### 2.1. The Study Population and Design

We performed a retrospective observational study of a cohort of patients in the biological therapy registry of the Rheumatology Department of Virgen Macarena University Hospital (HUVM), Seville, Spain. We included patients with RA, SpA, and PsA who had started treatment with targeted therapy between 2019 and late 2024. This study was approved by the Clinical Research Ethics Committee of HUVM (reference no., 0817-N-21).

### 2.2. The Protocol and Variables

The main outcome measures were MACEs, which included heart attack, stroke, and arterial or venous thrombosis. We recorded demographic variables such as age and sex, comorbidities associated with traditional cardiovascular risk factors (smoking, obesity, hypertension, diabetes mellitus, and personal history of heart disease), and the other comorbid conditions included in the Charlson comorbidity index. We recorded data on inflammation and calculated the 28-Joint Disease Activity Score with C-reactive protein (DAS28-CRP) and the Clinical Disease Activity Index (CDAI) for patients with RA; the Ankylosing Spondylitis Disease Activity Score with CRP (ASDAS-CRP) for patients with SpA; and the Disease Activity in Psoriatic Arthritis (DAPSA) scores for patients with PsA at the start of treatment, at the last check-up, and at the time of the event (where applicable). We also included severity-related measures such as the presence of structural damage, radiographic sacroiliitis, and extra-articular manifestations. We recorded all of the csDMARDs and/or bDMARDs that patients had received during the course of their disease and were receiving at the cut-off, as well as treatment with corticosteroids at the initiation of therapy and the dose administered.

### 2.3. The Statistical Analysis

First, we performed a descriptive analysis. Qualitative variables are expressed as absolute numbers and percentages and quantitative variables as the mean and standard deviation (SD). The χ^2^ test, the *t* test, and an analysis of variance were performed to compare the characteristics of the 3 disease groups and patients who experienced/did not experience a MACE. A multivariate logistic regression analysis was performed to identify the independent risk factors associated with the onset of a MACE. Variables that proved to be statistically significant in the bivariate analysis or that were of clinical interest were included in the model (using the combined forced-entry and backward stepwise method). The *p* value was set at <0.05 for all of the analyses, which were performed using R 2.4-0.

## 3. Results

### 3.1. General Characteristics

The study population comprised 137 patients (RA, 110: SpA, 9; and PsA, 18). The baseline characteristics (i.e., at the initiation of therapy with JAK inhibitors) of all three groups are shown in Table 1. Patients with RA were generally older, mainly female, and more frequently smokers. They were also characterized by more frequent dyslipidemia and had higher Charlson comorbidity indexes.

**Figure 1:**
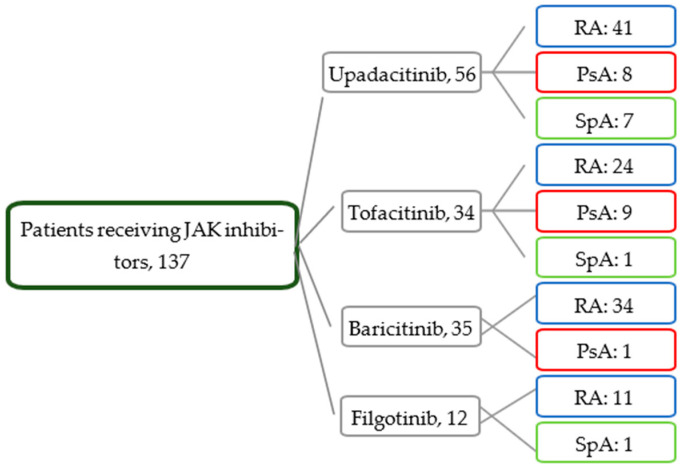
Distribution of patients by treatment and disease.

As for treatment, the patients with RA more frequently took corticosteroids and at greater doses. At the initiation of treatment with JAK inhibitors, the patients from all three groups had moderate–high disease activity. Figure 1 shows the distribution of the JAK inhibitors.

Most patients had received JAK inhibitors as the second-line or subsequent treatment. JAK inhibitors were the first-line therapy in only 20% of the patients (n = 28).

### 3.2. The Follow-Up

Patients were followed up for a mean of 3.9 (2.6) years. Two-thirds (82 [66.7%]) continued to receive treatment at the end of this period. Table 2 shows the efficacy data. At the end of the follow-up, a significant reduction was found in the DAS28, DAPSA score, and ASDAS values. The doses of corticosteroids fell significantly. In addition, 47.7% of patients who initiated JAK inhibitor therapy suspended it, 36 patients suspended csDMARD use, and 12 patients were taking optimized doses of JAK inhibitors. The optimization was greater in patients with RA (*p* < 0.001).

A total of 51 patients discontinued treatment after a mean of 2.4 (2.5) years. The main cause was secondary failure (16 patients), followed by primary failure (8), cancer (6), respiratory/urinary infections (7), acute myocardial infarction (1), cerebrovascular accidents (2), deep vein thrombosis (1), and other (the wish to become pregnant, gastrocnemius hematoma, epigastric pain, kidney failure, or elevated transaminases).

### 3.3. Adverse Events

Nine patients (6.6%) had experienced a MACE by the end of the follow-up. Table 3 shows the adverse events by drug. No significant differences were observed.

All of the patients who had experienced a MACE had RA (no MACEs were recorded in the patients with PsA or SpA) (Table 4). One patient died in the baricitinib group because of the progression of pancreatic cancer, and another died in the tofacitinib group because of the progression of melanoma. Both had RA. The remaining cancers detected were breast cancer (two patients), ovarian cancer (one patient), laryngeal cancer (one patient), cervical cancer (one patient), melanoma (one patient), and nonmelanoma skin cancer (two patients).

The characteristics of the patients who experienced MACEs are shown in Table 4.

When the patients with RA who did not experience MACEs were compared with the nine patients who had, we found that the former were more frequently male, smokers (current or previous), hypertensive, and diabetic. No significant differences were found with respect to disease activity or concomitant or previous treatment (Table 5). All of the patients who experienced MACEs received treatment for their cardiovascular comorbidity (diabetes mellitus, hypertension, and/or dyslipidemia), except for one patient who experienced an acute myocardial infarction and did not receive lipid-lowering therapy.

The logistic regression analysis of the RA patients showed that the factors independently associated with MACEs were a previous cardiovascular event (OR, 10.74; 95%CI, 1.05–113.7; *p* = 0.036), male sex (OR, 9.7; 95%CI, 1.6–76.5; *p* = 0.016), diabetes mellitus (OR, 10.3; 95%CI, 1.75–83; *p* = 0.013), and the duration of JAK inhibitor therapy (OR, 1.47; 95%CI, 1.13–2.01; *p* = 0.005).

## 4. Discussion

Our study presents the real-world efficacy and safety outcomes for JAK inhibitors in their different indications from a tertiary hospital’s registry. Several meta-analyses have been published since the alert raised by the PRAC [12,16], and while they have included results from clinical trials with these agents, the real-world safety data remain unclear.

Patients with RA, SpA, and PsA are at greater cardiovascular risk than healthy individuals. Therefore, we might expect to find a higher incidence of MACEs in this population [1,3]. However, we only found MACEs in patients with RA, consistent with the data from other registries—for example, the Spanish registry BIOBADASER [17]. As reported elsewhere [18], the most common presentations were heart attack and stroke. Compared with the patients affected by SpA and PsA, those with RA were older and more frequently smokers, with more comorbid conditions and a longer time since diagnosis. In addition, they more frequently took corticosteroids. The association between the use of corticosteroids and increased cardiovascular risk is controversial [19]. While our study revealed that the consumption of corticosteroids was more frequent in the patients who experienced MACEs, we found no significant differences for these drugs when we compared patients who experienced MACEs with those who did not.

A recent meta-analysis [12] of 39 clinical trials (16,894 participants) revealed that upadacitinib was the only JAK inhibitor to be significantly associated with an increased frequency of adverse events. However, the short duration of these trials made it impossible to draw firm conclusions on long-term safety. In our study, we found no significant differences between the four JAK inhibitors evaluated with respect to adverse events.

Along these lines, data published in October 2023 from an analysis performed as part of the German registry RABBIT [20] revealed MACEs in a large cohort of RA patients treated with bDMARDs, JAK inhibitors, and csDMARDs. The authors found no increase in the risk of adverse events in patients receiving JAK inhibitors compared with that under other treatments or differences between the JAK inhibitors. These findings are consistent with those of other studies [21] and ours and contrast with those of the ORAL Surveillance study [22]. Similarly, the authors did not find a higher risk of MACEs in patients aged > 65 years, patients with previous adverse events, or in smokers. In our study, the risk of experiencing a MACE did in fact increase in patients who had already experienced a cardiovascular event. However, the multivariate analysis revealed no association with age or smoking.

Although tobacco use did not show significant differences in the multivariate analysis, when we compared patients who had experienced a MACE with those who had not, we did see that those who experienced a MACE were more often smokers. Furthermore, compared to the rest of the traditional cardiovascular risk factors, we also found more diabetic and hypertensive patients in the MACE group in the univariate analysis, with the significant relationship with diabetes mellitus confirmed in the multivariate analysis. These patients received treatment for these comorbidities, although we do not know whether these were adequately controlled. It is well known that the increased cardiovascular risk in our patients is due to both traditional and non-traditional cardiovascular risk factors [6,7,8]. Therefore, in addition to strict disease control, we should be more aware of these modifiable traditional cardiovascular risk factors and act accordingly. For years, the European guidelines have recommended that rheumatologists be the primary caregivers for these factors [23]; however, this practice is not yet fully implemented. Perhaps we should consider making greater use of multidisciplinary consultations, which are becoming more common every day, with specialists such as pneumologists for quitting smoking, endocrinologists for diabetes management, and cardiologists for cardiovascular risk assessments.

Furthermore, we included several patients with previous cardiovascular events, an age > 65 years, and, as previously mentioned, with CVRFs because many of these treatments were started before the EMA’s alert or because there were no further therapeutic alternatives since these patients were refractory to other drugs. The patients were fully informed about the alert; after providing their consent, most continued treatment since their disease was controlled or they had no other treatment options available. While our study was not designed to evaluate the impact of the EMA’s alert, we consider its impact to be minor, consistent with a Spanish study that did analyze this alert [21]. More studies are necessary to clarify the risk of MACEs with these therapies. However, the treatment must be tailored and discussed with the patient to ensure a personalized approach.

The risk of cardiovascular events is one of the most concerning adverse effects. However, the most common adverse effect was infection. In our study and elsewhere [23], the most frequent infections were upper and lower airway infections and urinary tract infections, with similar incidence rates for the different JAK inhibitors. A recent large meta-analysis concluded that filgotinib use involved a significantly lower risk of infections than that of the other jakinibs, whereas baricitinib use involved a significantly higher risk of herpes zoster [12]. In our study, the number of patients who developed herpes zoster was higher in patients receiving tofacitinib and baricitinib, although this difference was not significant. The latest recommendations from the Spanish Society of Rheumatology [24,25] recommend vaccination with a recombinant herpes zoster vaccine in these patients receiving targeted therapies, so a future decrease in these types of infections is expected.

As for the risk of cancer, our results suggest that malignancy is more frequent with tofacitinib and baricitinib than with other JAK inhibitors, and while this difference is not statistically significant, it does tend toward significance. Nevertheless, we are unable to draw robust conclusions in this respect, and more studies should be performed to evaluate cancer in patients receiving JAK inhibitors. A meta-analysis designed to evaluate the risk of cancer with tofacitinib compared to that with other biologics concluded that tofacitinib did not increase the risk of malignancy; however, given the serious nature of this problem, continuous pharmacovigilance is necessary in the patients we treat [26]. A recent study concluded that there was a non-significant overall increase in cancer with these therapies. Upadacitinib presented the lowest risk of malignancy and baricitinib the highest, although neither finding was significant [12], results consistent with ours.

As for efficacy, our findings agree with those reported in clinical trials [27,28,29,30]. At the end of the follow-up, we recorded a significant reduction in disease activity for all three conditions, as well as a decrease in acute-phase reactant values. The corticosteroid doses fell significantly, and 47.7% of patients who took these agents at the initiation of JAK inhibitor therapy discontinued them.

Our study is subject to a series of limitations. First, its design was retrospective. However, the main part was prospective since all of the variables analyzed were recorded prospectively and systematically—hence, the total availability of the data and the consistent results. Moreover, our follow-up, of almost 4.5 years, is longer than that in other studies [17] and clinical trials. Second, the number of patients for each disease and drug was low, thus affecting the statistical power. However, all patients were treated under real-world conditions, and in contrast with other studies, we included all four JAK inhibitors currently available for rheumatic disease. Lastly, we did not compare the safety results with those for patients receiving biologics such as TNF-α or IL-6 inhibitors, thus necessitating further studies.

In conclusion, our results demonstrate the efficacy of JAK inhibitors in various rheumatic diseases and are similar to those reported elsewhere. We found no differences between the drugs in terms of adverse events. Specifically, we found no significant differences for MACEs. These events are more common in patients with CVRFs such as diabetes mellitus or those who have already experienced a cardiovascular event. JAK inhibitors are beneficial for disease control since they enable the broad suppression of cytokines, thus helping patients who are refractory to other treatments and adding the advantage of oral administration. Notwithstanding, their long-term safety should continue to be evaluated in real-world studies.

## Figures and Tables

**Table 1:** Baseline patient data.

Variable	Rheumatoid Arthritis (n = 110)	Spondyloarthritis (n = 9)	Psoriatic Arthritis (n = 18)	*p* Value
**Baseline characteristics**				
Age, years, mean (SD)	58.2 (12.5)	43.6 (14.8)	49.9 (12)	**<0.001**
Female sex, n (%)	89 (80.9)	4 (44.4)	12 (66.7)	**0.025**
Smoking				**<0.001**
Ex-smoker, n (%)	12 (10.9)	1 (11.1)	1 (5.6)	**<0.001**
Active smoker, n (%)	23 (20.9)	3 (33.3)	2 (11.1)	**<0.001**
**Comorbidities**				
Hypertension, n (%)	44 (40)	2 (22.2)	4 (22.2)	0.228
Diabetes mellitus, n (%)	5 (3.8)	1 (0.6)	1 (2.5)	0.222
Dyslipidemia, n (%)	46 (31.1)	1 (11.1)	3 (7.5)	**<0.001**
Obesity, n (%)	20 (18.2)	1 (11.1)	1 (5.6)	**0.367**
Previous CV events, n (%)	10 (8.2)	0	1 (5.6)	0.632
Heart attack, n (%)	4 (3.6)	0	0	0.603
Stroke, n (%)	4 (3.6)	0	1 (5.6)	0.768
Age-adjusted Charlson index, mean (SD)	3.1 (2.02)	1.4 (1.01)	2.05 (1.8)	**0.007**
**Disease characteristics**				
Time since diagnosis (years), mean (SD)	20.1 (11.4)	11.8 (7.6)	16.2 (9.29)	**0.048**
Structural damage, n (%)	57 (51.8)	2 (22.2)	4 (22.2)	0.057
Rheumatoid factor-positive, n (%)	96 (87.3)	-	-	-
ACPA-positive, n (%)	96 (87.3)	-	-	-
Baseline DAS28-CRP, mean (SD)	4.95 (1.1)	-	-	-
Baseline CRP (mg/dl), mean (SD)	20.1 (20.8)	20.5 (18)	8.2 (9.3)	0.151
CDAI at cut-off, mean (SD)	26.1 (14.4)	-	-	-
HLAB27-positive, n (%)	-	5 (55.5)	2 (11.1)	-
Axial SpA, n (%)	-	3 (33.3)	-	-
Radiographic sacroiliitis, n (%)	-	6 (66.7)	2 (11.1)	-
ASDAS-CRP, mean (SD)	-	3.6 (0.5)	-	-
DAPSA score, mean (SD)	-	-	41.3 (28.6)	-
Moderate–high disease activity, n (%)	92 (83.6)	6 (66.7)	14 (77.7)	**-**
**Treatment**				
Previous bDMARD, n (%)	86 (78.2)	7 (77.8)	15 (33.3)	0.088
Concomitant csDMARD, n (%)	58 (52.7)	2 (22.2)	7 (38.9	0.140
Methotrexate, n (%)	45 (77.6)	2 (100)	7 (100)	**-**
Corticosteroids, n (%)	83 (72.7)	1 (11.1)	7 (38.9)	**<0.001**
Corticosteroid dose at cut-off, mean (SD)	6.3 (6.7)	1.1 (2.2)	3.3 (7.1)	**0.025**

Abbreviations: CV, cardiovascular; ACPA, anti-citrullinated peptide antibody; DAS28, 28-Joint Disease Activity Score; CRP, C-reactive protein; CDAI, Clinical Disease Activity Index; ASDAS, Ankylosing Spondylitis Disease Activity Score; DAPSA, Disease Activity in Psoriatic Arthritis; bDMARD, biologic disease-modifying antirheumatic drug; csDMARD, conventional synthetic disease-modifying antirheumatic drug. Numbers in bold and red color are statistically significant.

**Table 2:** Efficacy data at end of follow-up.

Variable		Rheumatoid Arthritis (n = 110)	Spondyloarthritis (n = 9)	Psoriatic Arthritis (n = 18)	*p* Value
Follow-up (years), mean (SD)		4.04 (2.6)	2.1 (1.05)	4.1 (2.9)	0.095
Time receiving JAK inhibitors (years), mean (SD)		3.4 (2.7)	1.2 (0.8)	2.9 (2.8)	0.062
DAS28-CRP, mean (SD)	Baseline	4.95 (1.1)	-	-	
	End	2.3 (0.8)	-	-	
ASDAS-CRP, mean (SD)	Baseline	-	3.6 (0.5)	-	
	End	-	2.1 (0.6)	-	
DAPSA score, mean (SD)	Baseline	-	-	41.3 (28.6)	
	End	-	-	9.2 (8.1)	
CRP, mean (SD)	Baseline	20.1 (20.8)	20.5 (18)	8.2 (9.3)	0.151
	End	7.5 (12.8)	4.4 (7.5)	6.2 (6.9)	0.832
Remission-low activity, n (%)		60 (54.5)	4 (44.4)	9 (50)	**-**
Corticosteroids, n (%)		19 (17.3)	0	0	0.064
Corticosteroids, mean (SD)	Baseline	6.3 (6.7)	1.1 (2.2)	3.3 (7.1)	**0.025**
	End	1.3 (2.7)	0	0	0.168
Corticosteroids discontinued, n (%)		36 (32.7)	1 (11.1)	5 (27.7)	0.222
csDMARDs discontinued, n (%)		32 (29.1)	0	4 (22.2)	0.156
Optimization, n (%)		10 (9.1)	0	2 (11.1)	**<0.001**

Abbreviations: DAS28, 28-Joint Disease Activity Score; CRP, C-reactive protein; ASDAS, Ankylosing Spondylitis Disease Activity Score; DAPSA, Disease Activity in Psoriatic Arthritis; csDMARD, conventional synthetic disease-modifying rheumatic drug. Numbers in bold are statistically significant.

**Table 3:** Adverse events.

Adverse Event	Upadacitinib (n = 56)	Tofacitinib (n = 34)	Baricitinib (n = 35)	Filgotinib (n = 12)	*p* Value
MACEs, n (%)	1 (0.9)	4 (11.7)	4 (11.4)	0	0.123
Heart attack, n (%)	1 (0.9)	3 (8.8)	1 (2.9)	0	0.644
Stroke, n (%)	0	1 (2.9)	2 (5.8)	0	0.308
Thrombosis, n (%)	0	0	1 (2.9)	0	0.401
Neoplasm, n (%)	1 (0.9)	5 (14.7)	4 (11.4)	0	0.068
Herpes zoster, n (%)	3 (2.7)	3 (8.8)	3 (8.5)	0	0.621
Other infections, n (%)	17 (15.5)	13 (38.2)	12 (34.2)	2 (16.7)	0.561

Abbreviations: MACE, major acute cardiovascular event.

**Table 4:** Characteristics of patients who developed MACEs.

Patient	MACE	Age (Years) at Initiation of JAK Inhibitor	Sex	CVRFs	Treatment for CVRFs at Event	JAK Inhibitor	Time Receiving JAK Inhibitor at Event (Years)	CRP (mg/dl)	DAS28 at Event
**1**	AMI	49	Male	Smoking, HT, pericarditis	Yes	Tofacitinib	3.7	30	4.63
**2**	AMI	72	Female	HT, DM, DL	Yes	Tofacitinib	6.6	1.2	2.08
**3**	AMI	51	Female	Ex-smoker, HT, DM, DL	Yes	Tofacitinib	0.8	6.7	3.09
**4**	CVA	65	Male	None	---	Tofacitinib	5.4	3.6	1.75
**5**	AMI	82	Female	HT, DM, previous AMI	Yes	Upadacitinib	2.7	1.8	5.41
**6**	AMI	60	Male	DL	No	Baricitinib	1.1	116	7.7
**7**	DVP	58	Female	Smoking, HT	Yes	Baricitinib	6.8	16	2.27
**8**	CVA	84	Female	HT, DM, pacemaker, and aortic prosthesis	Yes	Baricitinib	2.5	17	2
**9**	CVA	59	Male	Ex-smoker, HT	Yes	Baricitinib	3.7	4	1.54

Abbreviations: AMI, acute myocardial infarction; CVA, cerebrovascular accident; DVP, deep venous thrombosis; HT, hypertension; DM, diabetes mellitus; DL, dyslipidemia; CVRF, cardiovascular risk factor; CRP, C-reactive protein; DAS28, 28-Joint Disease Activity Score.

**Table 5:** Patients who experienced and did not experience MACEs.

Variable	No MACE (n = 101)	MACE (n = 9)	*p* Value
**Baseline characteristics**			
Age (years), mean (SD)	57.7 (12.4)	64.7 (12.5)	0.137
Male sex, n (%)	17 (16.8)	4 (44.4)	**0.043**
Smoking			
Ex-smoker, n (%)	10 (9.9)	2 (22.2)	**0.020**
Active smoker, n (%)	21 (20.7)	2 (22.2)	**<0.001**
**Comorbidities**			
Hypertension, n (%)	37 (36.6)	7 (77.7)	**0.015**
Diabetes mellitus, n (%)	14 (13.8)	4 (44.4)	**0.017**
Dyslipidemia, n (%)	40 (39.6)	3 (33.3)	0.718
Obesity, n (%)	17 (16.8)	3 (33.3)	0.218
Previous CV events, n (%)	7 (6.9)	2 (22.2)	0.108
Age-adjusted Charlson comorbidity index, mean (SD)	3.04 (1.9)	4.3 (2.2)	0.121
**Disease characteristics**			
Time since diagnosis (years), mean (SD)	20.2 (11.5)	19.3 (10.2)	0.808
Structural damage, n (%)	52 (51.4)	5 (55.5)	0.837
Rheumatoid factor-positive, n (%)	90 (89.1)	6 (66.6)	0.344
ACPA-positive, n (%)	89 (88.1)	7 (77.7)	0.896
Baseline DAS28-CRP, mean (SD)	4.9 (1.1)	5.4 (0.9)	0.177
Baseline CRP (mg/dl), mean (SD)	19.2 (20.8)	34.6 (15.8)	0.094
CDAI at cut-off, mean (SD)	26.1 (14.6)	27 (11.3)	0.888
**Treatment**			
Previous bDMARDs, n (%)	81 (80.2)	5 (55.5)	0.086
≥3 previous biologics, n (%)	41 (40.6)	3 (33.3)	0.670
Time on JAK inhibitors (years), mean (SD)	3.2 (2.7)	5.7 (2)	**0.004**
Concomitant csDMARDs, n (%)	54 (53.5)	4 (44.4)	0.603
Methotrexate, n (%)	42 (41.5)	5 (55.5)	0.795
Corticosteroids, n (%)	73 (72.3)	7 (77.7)	0.177

Abbreviations: CV, cardiovascular; ACPA, anti-citrullinated peptide antibody; DAS28, 28-Joint Disease Activity Score; CRP, C-reactive protein; CDAI, Clinical Disease Activity Index; bDMARD, biologic disease-modifying antirheumatic drug; csDMARD, conventional synthetic disease-modifying antirheumatic drug. Numbers in bold and red color are statistically significant.

## Data Availability

The original contributions presented in this study are included in the article. Further inquiries can be directed to the corresponding author.
